# Errata

**Published:** 2015-04-03

## Vol. 64, No. 1

In the report, “Incidence of Notifiable Diseases Among American Indian/Alaska Natives — United States, 2007–2011,” multiple errors occurred in the text and table.

In the third paragraph, the first sentence should read “For 26 notifiable diseases examined for 2007–2011, a total of **9,061,675** cases were recorded (Table).” The third sentence should read, “Missing data on race ranged from 0.8% for tuberculosis to 42% for giardiasis.”

In the fourth paragraph, the first sentence should read, “Of the 12 diseases with race information for >70% of records and for which rates were higher among AI/ANs than among whites, the largest difference was for hantavirus pulmonary syndrome, which was reported **10** times more often among AI/ANs than among whites; however, only 20 cases were reported among AI/ANs of a total of 112 cases reported during 2007–2011.” The second sentence should read, “The second largest difference was for tularemia, which was reported **7.7** times as often among AI/ANs.”

On page 17, the Table should have read as follows:

**TABLE:** Number and incidence rate per 100,000 population for 26 selected notifiable diseases, by American Indian/Alaska Native (AI/AN), black, or white race — United States, 2007–2011

Disease	AI/ANs		Blacks		Whites		Total		Rate ratio: AI/ANs compared with whites	% with no race identified
			
	No.	Rate	No.	Rate	No.	Rate	No.	Rate		
Botulism, foodborne	26	0.12	28	**0.01**	339	0.03	672	0.04	4.38	35.42
Chickenpox (varicella)	503	2.45	7,086	**3.41**	78,776	6.45	110,634	7.22	0.38	17.82
					78,776					
*Chlamydia trachomatis*	**77,072**	**374.83**	2,189,748	**1,052.68**	1,841,172	150.74	6,283,761	409.90	2.49	**29.84**
Cryptosporidiosis	223	1.08	3,202	**1.54**	29,010	2.38	45,721	2.98	0.46	25.63
Ehrlichiosis, total	219	1.07	167	**0.08**	7,250	0.59	12,348	0.81	1.79	36.46
Gonorrhea	12,764	62.08	894,198	**429.87**	317,271	25.97	**1,625,097**	**106.01**	2.39	**21.70**
Giardiasis	385	1.87	6,875	**3.31**	38,506	3.15	93,164	6.08	0.59	41.55
*Haemophilus influenzae*	206	1.00	1,822	**0.88**	9,340	0.76	14,990	0.98	1.31	20.69
Hantavirus pulmonary syndrome	20	0.10	1	**0.00**	77	0.01	112	0.01	15.43	10.71
Hepatitis A, viral acute	66	0.32	677	**0.33**	5,607	0.46	10,544	0.69	0.70	28.15
Hepatitis B, viral acute	144	0.70	3,532	**1.70**	9,433	0.77	**18,114**	**1.18**	0.91	2.22
Hepatitis C, viral acute	88	0.43	261	**0.13**	3,220	0.26	4,553	0.30	1.62	19.33
Legionellosis	42	0.20	2,890	**1.39**	10,590	0.87	16,870	1.10	0.24	16.87
Lyme disease	476	2.31	1,649	**0.79**	85,721	7.02	160,209	10.45	0.33	38.68
Meningococcal disease	48	0.23	707	**0.34**	2,899	0.24	4,776	0.31	0.98	18.91
Pertussis	788	3.83	3,709	**1.78**	57,644	4.72	85,723	5.59	0.81	23.48
Salmonellosis	1,783	8.67	21,647	**10.41**	142,495	11.67	252,169	16.45	0.74	28.99
Shiga toxin–producing *Escherichia coli*	161	0.78	1,020	**0.49**	16,749	1.37	26,058	1.70	0.57	27.09
Shigellosis	1,115	5.42	17,822	**8.57**	37,309	3.05	85,172	5.56	1.78	28.71
Spotted fever rickettsiosis	519	2.52	434	**0.21**	7,325	0.60	11,108	0.72	4.21	23.17
*Streptococcus pneumoniae*, invasive (all ages)	575	2.80	8,652	**4.26**	28,766	2.36	49,548	3.23	1.19	20.76
*Streptococcus pneumoniae*, invasive (age <5 years)	297	**15.92**	2,249	**13.38**	9,214	**12.04**	16,102	**15.95**	**1.32**	21.96
Syphilis, primary and secondary	367	1.78	31,469	**15.13**	**28,616**	2.30	66,707	4.35	0.78	4.00
Tuberculosis	813	3.95	15,167	**7.29**	25,944	2.12	59,458	3.88	1.86	0.77
Tularemia	47	0.23	15	**0.01**	413	0.03	626	0.04	6.76	21.73
West Nile virus disease	184	0.89	348	**0.17**	5,142	0.42	**7,439**	**0.49**	2.13	**21.86**
**Total**	**98,931**	**—**	**3,215,375**	**—**	**2,798,828**	**—**	**9,061,675**	**—**	**—**	**—**

## Vol. 64, No. 6

In the report, “Update on Progress in Selected Public Health Programs After the 2010 Earthquake and Cholera Epidemic — Haiti, 2014,” in the Figure on page 139, the data shown for “Eligible children receiving measles-rubella vaccination” pertain only to estimated coverage through routine immunization. Additional vaccinations were provided through special campaigns.

